# Comparison of conventional MRI analysis versus MRI-based radiomics to predict the circumferential margin resection involvement of rectal cancer

**DOI:** 10.1186/s12876-024-03274-z

**Published:** 2024-06-20

**Authors:** Hong Liang, Dongnan Ma, Yanqing Ma, Yuan Hang, Zheng Guan, Yang Zhang, Yuguo Wei, Peng Wang, Ming Zhang

**Affiliations:** 1 Department of Medical Imaging, The First Affiliated Hospital of Xi’an Jiaotong University, No. 277, Yanta West Road, Xi’an, 710061 China; 2 School of Medical Imaging, Hangzhou Medical College, No.481, Binwen Road, Hangzhou, 310000 China; 3 Yangming College of Ningbo University, Ningbo, China; 4 Department of Radiology, Zhejiang Provincial People’s Hospital, Affiliated People’s Hospital of Hangzhou Medical College, Hangzhou, China; 5 Department of Colorectal Surgery, Zhejiang Provincial People’s Hospital, Affiliated People’s Hospital of Hangzhou Medical College, Hangzhou, China; 6 GE Healthcare, Precision Health Institution, Hangzhou, China; 7 Department of Radiology, 411 Hospital of Shanghai University, No.15, Dongjiangwan Road, Shanghai, 200080 China

**Keywords:** Rectal cancer, Magnetic resonance imaging, Mesorectal fascia, Radiomics

## Abstract

**Background:**

To compare the application of conventional MRI analysis and MRI-based radiomics to identify the circumferential resection margin (CRM) status of rectal cancer (RC).

**Methods:**

A cohort of 301 RC patients with 66 CRM invloved status and 235 CRM non-involved status were enrolled in this retrospective study between September 2017 and August 2022. Conventional MRI characteristics included gender, age, diameter, distance to anus, MRI-based T/N phase, CEA, and CA 19 − 9, then the relevant logistic model (Logistic-cMRI) was built. MRI-based radiomics of rectal cancer and mesorectal fascia were calculated after volume of interest segmentation, and the logistic model of rectal cancer radiomics (Logistic-rcRadio) and mesorectal fascia radiomics (Logistic-mfRadio) were constructed. And the combined nomogram (nomo-cMRI/rcRadio/mfRadio) containing conventional MRI characteristics, radiomics of rectal cancer and mesorectal fascia was developed. The receiver operator characteristic curve (ROC) was delineated and the area under curve (AUC) was calculated the efficiency of models.

**Results:**

The AUC of Logistic-cMRI was 0.864 (95%CI, 0.820 to 0.901). The AUC of Logistic-rcRadio was 0.883 (95%CI, 0.832 to 0.928) in the training set and 0.725 (95%CI, 0.616 to 0.826) in the testing set. The AUCs of Logistic-mfRadio was 0.891 (95%CI, 0.838 to 0.936) in the training set and 0.820 (95%CI, 0.725 to 0.905) in the testing set. The AUCs of nomo-cMRI/rcRadio/mfRadio were the highest in both the training set of 0.942 (95%CI, 0.901 to 0.969) and the testing set of 0.909 (95%CI, 0.830 to 0.959).

**Conclusion:**

MRI-based radiomics of rectal cancer and mesorectal fascia showed similar efficacy in predicting the CRM status of RC. The combined nomogram performed better in assessment.

**Supplementary Information:**

The online version contains supplementary material available at 10.1186/s12876-024-03274-z.

## Background

More than 0.73 million new rectal cancer (RC) cases and 339,000 deaths were estimated to occur in 2020, ranking about one in 10 cancer cases and deaths [1]. Total mesorectal excision (TME) has become the gold standard for curative resection of rectal cancer, and has shown universal reductions in local recurrence and improvement in disease-free survival [2]. The circumferential resection margin (CRM) status has been a significant indicator in evaluating the oncologic outcomes, the rates of local recurrence, distant metastasis, and overall survival following surgery, which is crucial for selection of postoperative adjuvant therapy [3]. CRM is composed of mesorectal fascia, enclosing the fatty mesorectum and rectum along with associated vessels, lymphatics, and lymph nodes. It is defined as the non-peritonealized surface of the resected specimen and arises during surgical mesorectal dissection of the inferior peritoneal surface [4]. Microscopic examination of the sections was conducted to assess the involvement of the lateral resection margin. The distance from the tumor extension or the outer border of mesorectal or the presence of a positive lymph node to the resection margin was measured, with a distance of less than or equal to 1 mm indicating potential CRM involvement [5]. Patients with involving CRM due to direct tumor spread have an even higher risk of local recurrence and poorer overall survival in comparison to those with lymph node spread [4].

Magnetic resonance imaging (MRI) is the most preferred diagnostic method for preoperative staging of RC, as recommended by guidelines. MRI excels in defining the mesorectal fascia and accurately predicting the tumor-free circumferential resection margin before total TME [6]. Studies on MRI evaluation of the minimal distance between tumor and mesorectal fascia showed that the accuracy for tumor-free CRM was 86.5% and the negative predict value was 98.1% [7]. Application of conventional MRI evaluation to aid in operative decision-making is difficult in the preoperative setting. Radiomics mines a large amount of quantitative data hidden in conventional radiological images with an aid of computer [8], and is gaining increasing traction in predicting RC outcomes. MRI-based radiomics is of paramount importance to RC, as it may help to predict the status of microsatellite instability [9], lymph node staging [10], extramural venous invasion [11], and so on. Nevertheless, the complementary performance of MRI-radiomics in assessing CRM status of RC, when combined with conventional MRI characteristics, remains an area of ongoing exploration.

To the best of our knowledge, there has been no published literature specifically examining the value of MRI-radiomics of mesorectal fascia and tumor in predicting the CRM status of RC. The purpose of our study is to explore the role of MRI-radiomics, along with conventional MRI characteristics, in evaluating the involved status of CRM.

## Methods

### Patient population

**Fig. 1:**
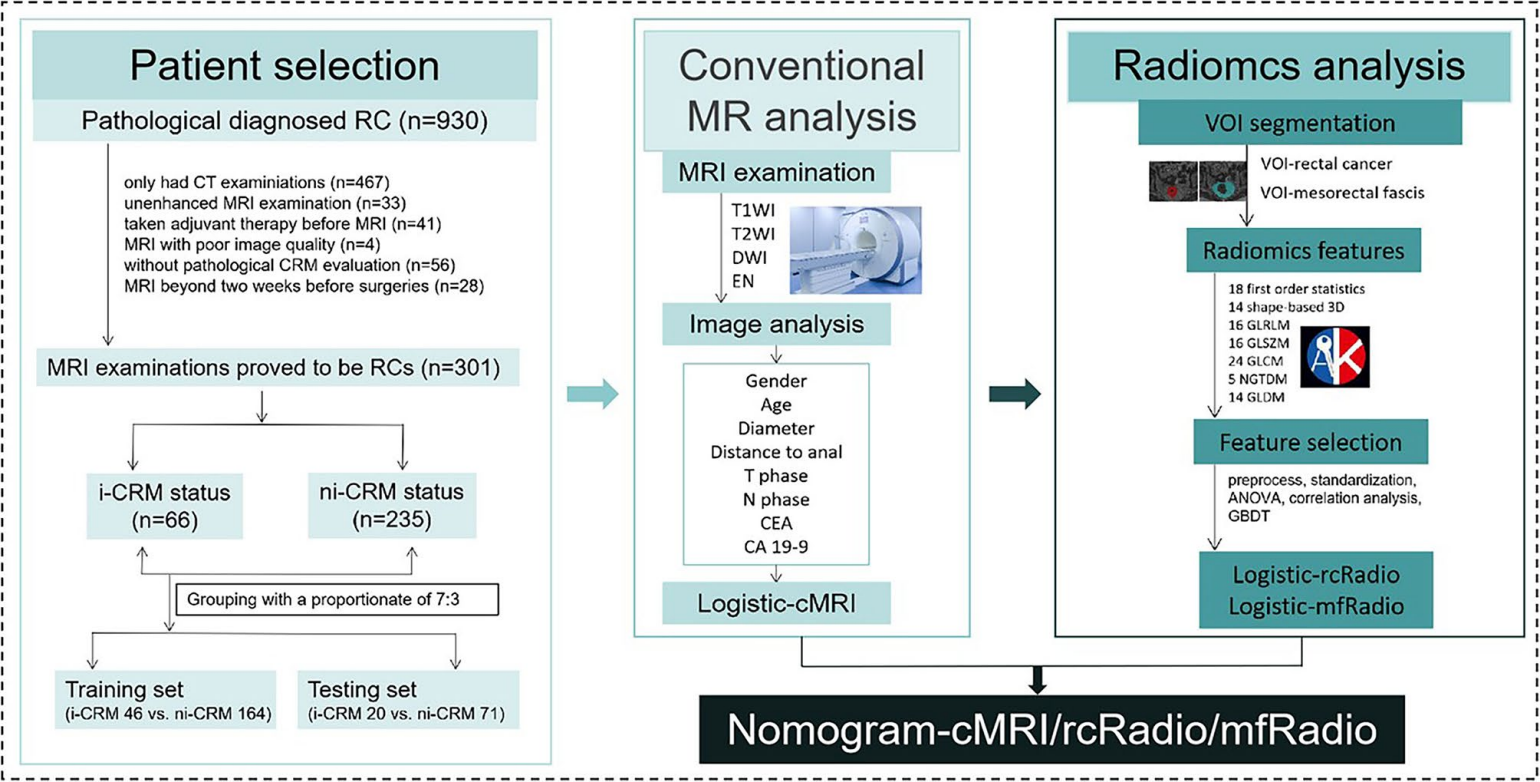
The general technical workflow of this study

This retrospective study was approved by the institutional research ethics committee (No. 2021QT339), and the informed consent was approved to be waived. There were 301 RC patients recruited, spanning from September 2017 to August 2022. The inclusion criteria were as follows: (a) patients were pathologically diagnosed as CRM involvement after surgeries; (b) surgeries were performed within two weeks after MRI; and (c) MRI sequences included axial T1-weighted imaging (T1WI), axial T2-weighted imaging (T2WI), axial diffusion-weighted imaging (DWI), and axial contrast-enhanced T1WI (T1CE). The exclusion criteria were as follows: (a) patients acquired adjuvant therapy, like chemotherapy, radiotherapy, or chemoradiotherapy before MRI and surgeries; (b) patients conducted unenhanced MRI; (c) the CRM status was not pathologically evaluated. Finally, the 301 patients were divided into two groups with 66 CRM involvement (i-CRM) and 235 without CRM involvement (ni-CRM) in this study. The general technical workflow was outlined in Fig. 1.

### MRI scan and analysis

All MRI scans were performed using a 3.0-T Discovery MR 750 scanner (GE Healthcare/Siemens Healthineers), utilizing an 8-channel phased-array coil in the supine position. The rectal MR examination encompassed multiple sequences: axial T1WI, axial and sagittal T2WI, axial DWI, ADC, and axial T1CE. The parameters were listed in Table 1. The Gadobenate dimeglumine was administered intravenously via an MRI-compatible power injector at a rate of 2mL/s and a dose of 0.2mL/kg, followed by a 20mL/s saline flush to complete the enhanced MRI. The sequences of axial T1WI, axial T2WI, axial DWI with b values of 0 and 1000s/mm2, and axial T1CE were chosen for analysis.

Conventional MR characteristics were collected by two radiologists with 10 and 12 years of MR diagnosis experience (Doctor Ma and Doctor Guan) in unanimity after discussion, containing gender, age, diameter of lesion, distance to anus, MR-based tumor stage (T1-4), MR-based lymph node stage (N0-2), carcinoma embryonic antigen (CEA), and carbohydrate antigen 19 − 9 (CA 19 − 9). A comprehensive list of clinical and MR information is listed in Table 2. The diagnosis criteria were listed in Supplementary Material-Sect. 1.

**Table 1:** The parameters of MRI scan

Sequence	TR (ms)	TE (ms)	FOV (cm)	Matrix	Thickness (mm)	Interval (mm)	B value (s/mm^2^)
T1WI	662	9.6	18*18	320*324	3.0	0.6	/
T2WI	4790	134	20*20	384*384	3.0	0.3–0.6	/
DWI	7330	56	20*20	112*101	3.0	0.8	0/1000
T1CE	616	9.6	18*18	224*320	3.0	0.6	/

### MRI-radiomics assessment

**Fig. 2:**
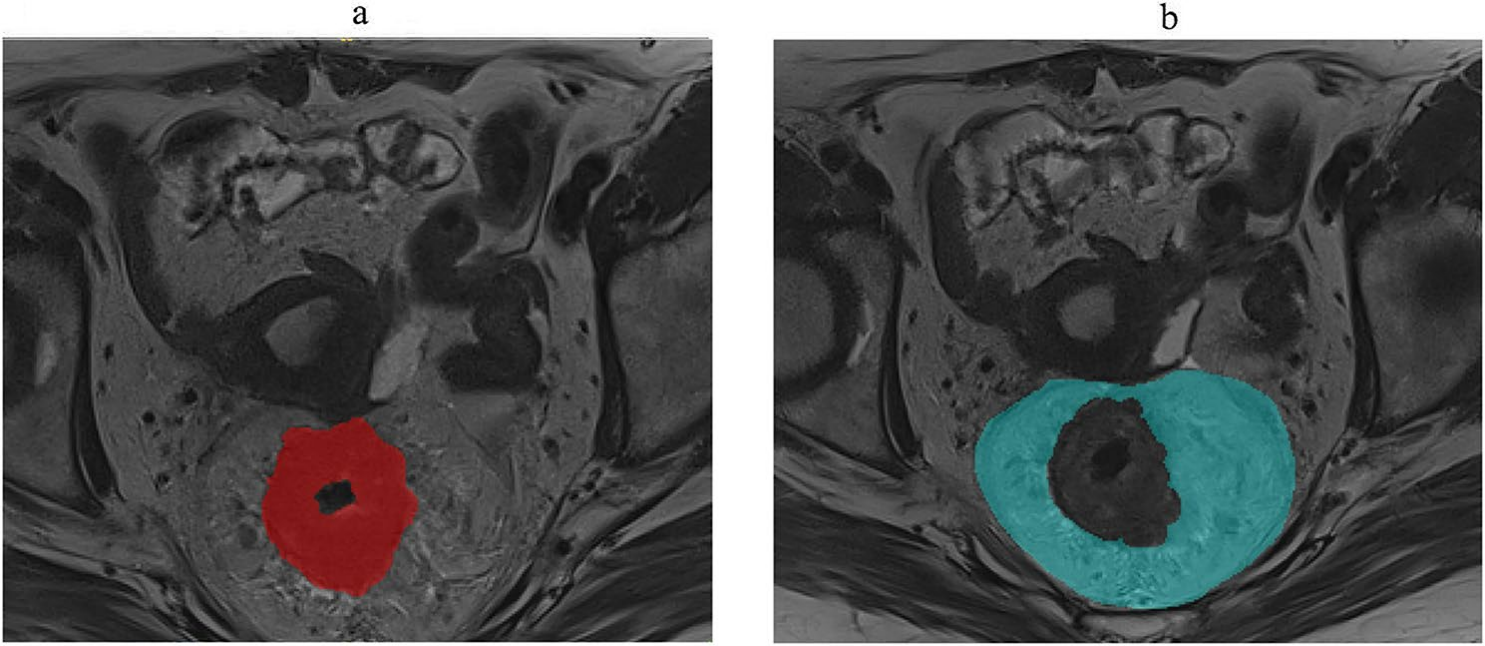
The segmentation of VOI-rc (**a**) and VOI-mf (**b**)

Volume of interest (VOI) segmentation: the VOI of rectal cancer (VOI-rc, Fig. 2a) and VOI of mesorectal fascia (VOI-mf, Fig. 2b) were manually delineated using software of ITK-SNAP (Version 3.8.0) in all of the T1WI, T2WI, DWI, and T1CE sequences with the help of Matlab software. The specific process of segmentation was detailed in Supplementary material-Sect. 2.

Then the radiomics features of rectal cancer and mesorectal fascia were calculated in the software of AK (GE Healthcare) after resampling the voxel of images into 1.0. There were 428 radiomics features from T1WI, T2WI, DWI, and T1CE sequences of rectal cancer and mesorectal fascia, individually. The specification of radiomics features were presented in Supplementary material-Sect. 3. Subsequently, four steps were applied to select optimal radiomics features: first, the method of synthetic minority over-sampling technique (SMOTE) was employed to address the class imbalance between i-CRM and ni-CRM sample sets. Second, the cohort was randomly divided into the training and testing set with a proportionate of 7:3. Third, outlier variables were replaced with median, and the method of standardization was applied. Fourth, the methods of analysis of variance, correlation analysis, and gradient boosting decision tree (GBDT) algorithm. were taken for dimension reduction. Finally, the optimal radiomics features were extracted for further analysis. Additional explanations regarding feature selection process was described in **Supplementary material-Sect. 4**.

### Model development

In the conventional MRI analysis, the significant variables constituted the logistic model (Logistic-cMRI) to predict the CRM status of RC. Parallel to this, the optimal radiomics features of rectal cancer and mesorectal fascia were employed to develop diagnostic models using a logistic regression approach. Specifically, two logistic models were constructed: one for MRI-based radiomics of rectal cancer (Logistic-rcRadio) and another for MRI-based radiomics of mesorectal fascia (Logistic-mfRadio), to predict the CRM status of RC. The corresponding receiver operator characteristic curve (ROC) was delineated, and the area under curve (AUC) was calculated to reckon the efficiency of these models.

### Statistics

The software of SPSS (Version 26) was taken to analyze the clinical and conventional MRI characteristics, by the methods of normality test, Manni Whitney u-test, and chi-square test. The approaches of feature reduction and model construction were performed by the software of Python (Version 3.5). The AUC curve was proceeded in MedCalc software (Version 15.8). A *p*-value less than 0.05 was considered statistically significant, indicating a meaningful difference between groups or a significant association between variables.

## Results

### Conventional MRI analysis

There were 301 RC patients recruited in this study, containing 66 i-CRM patients with mean age of 63.44 ± 13.06 years old and 235 ni-CRM patients with mean age of 64.57 ± 10.80 years old. Conventional MRI analysis revealed significant differences in characteristics of diameter, MRI-based T phase, MRI-based N phase, CEA, and CA 19 − 9 (Table 2). The diameter of RC with i-CRM status was larger than that of ni-CRM status (mean ± SD: 5.94 ± 2.21 vs. 4.84 ± 3.73). There were more patients in the i-CRM group who were tended to be in T4 phase (37.9% vs. 3.8%) and N2 phase (69.7% vs. 24.7%) compared to the ni-CRM group. Additionally, the levels of CEA (mean ± SD: 245.40 ± 1127.85 vs. 28.44 ± 311.44, µg/ml) and CA 19 − 9 (mean ± SD: 165.93 ± 868.95 vs. 20.08 ± 65.34, U/ml) of i-CRM group were significantly higher than those of ni-CRM group. A logistic regression with stepwise forward selection was then performed, resulting in the development of Logistic-cMRI model containing variables of MRI-based T phase, MRI-based N phase, and CA 19 − 9. The equation of Logistic-cMRI was: Y=-9.555 + 2.084*[MRI-based T phase] + 1.298*[MRI-based N phase] + 0.002*[CA 19 − 9]. The AUC of Logistic-cMRI was 0.864 (95%CI, 0.820 to 0.901).

**Table 2:** Conventional MRI characteristics

	ni-CRM (*n* = 235)		i-CRM (*n* = 66)		*p*
	Training set (164)	Testing set (71)	Training set (46)	Testing set (20)	
Gender (n)					0.37
Female	56	24	37	11	
Male	108	47	9	9	
Age (mean ± SD)	63.47 ± 11.32	67.10 ± 9.05	64.11 ± 11.67	61.90 ± 16.05	0.67
Diameter (cm)	5.00 ± 4.26	4.47 ± 2.01	6.05 ± 2.33	5.69 ± 1.93	<0.05
Distance to anus (cm)	7.42 ± 2.78	7.10 ± 2.82	6.73 ± 3.14	7.11 ± 3.22	0.14
MRI-based T phase (n)					<0.05
T1	2	1	0	0	
T2	41	14	0	0	
T3	116	52	31	10	
T4	5	4	15	10	
MRI-based N phase (n)					<0.05
N0	53	27	0	0	
N1	71	26	18	2	
N2	40	18	28	18	
CEA (µg/ml)	36.35 ± 372.49	10.16 ± 25.73	181.11 ± 785.95	393.28 ± 1689.87	<0.05
CA 19 − 9 (U/ml)	20.94 ± 75.90	18.09 ± 29.17	70.87 ± 204.93	384.59 ± 1552.89	<0.05

## MRI-based radiomics analysis of RC and mesorectal fascia

For the MRI-based radiomics analysis of rectal cancer, six radiomics features were left to form the Logistic-rcRadio. The AUC of this model was 0.883 (95%CI, 0.832 to 0.923) in the training set and 0.725 (95%CI, 0.621 to 0.813) in the testing set. To the entirety, the AUC was 0.835 (95%CI, 0.789 to 0.876), and the sensitivity was 71.2%, and the specificity was 83.0%. Then the corresponding radiomics score (rc-Radscore) was calculated.

For the MRI-based radiomics analysis of mesorectal fascia, the remaining 7 radiomics features constructed the Logistic-mfRadio. The AUC of this model in the training set was 0.891 (95%CI, 0.841 to 0.930) and in the testing set was 0.820 (95%CI, 0.726 to 0.893). When considering the entire cohort, the AUC was 0.871 (95%CI, 0.827 to 0.906), and the sensitivity was 77.3%, and the specificity was 83.0%. Then the corresponding radiomics score (mf-Radscore) was calculated.

## Nomogram combined conventional MRI and radiomics analysis

**Fig. 3:**
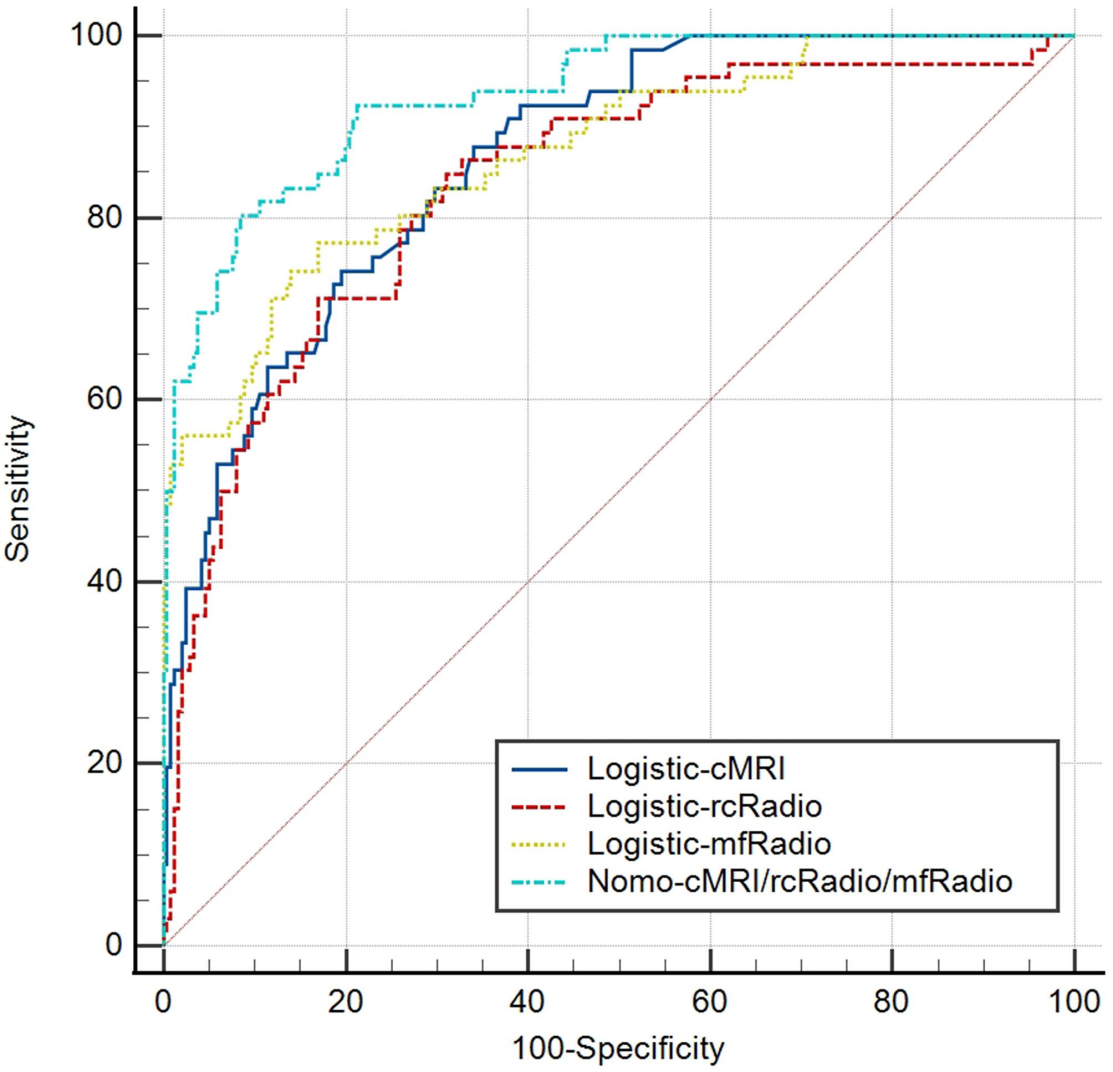
The comparison of ROC of overall cohort of predictive models

**Fig. 4:**
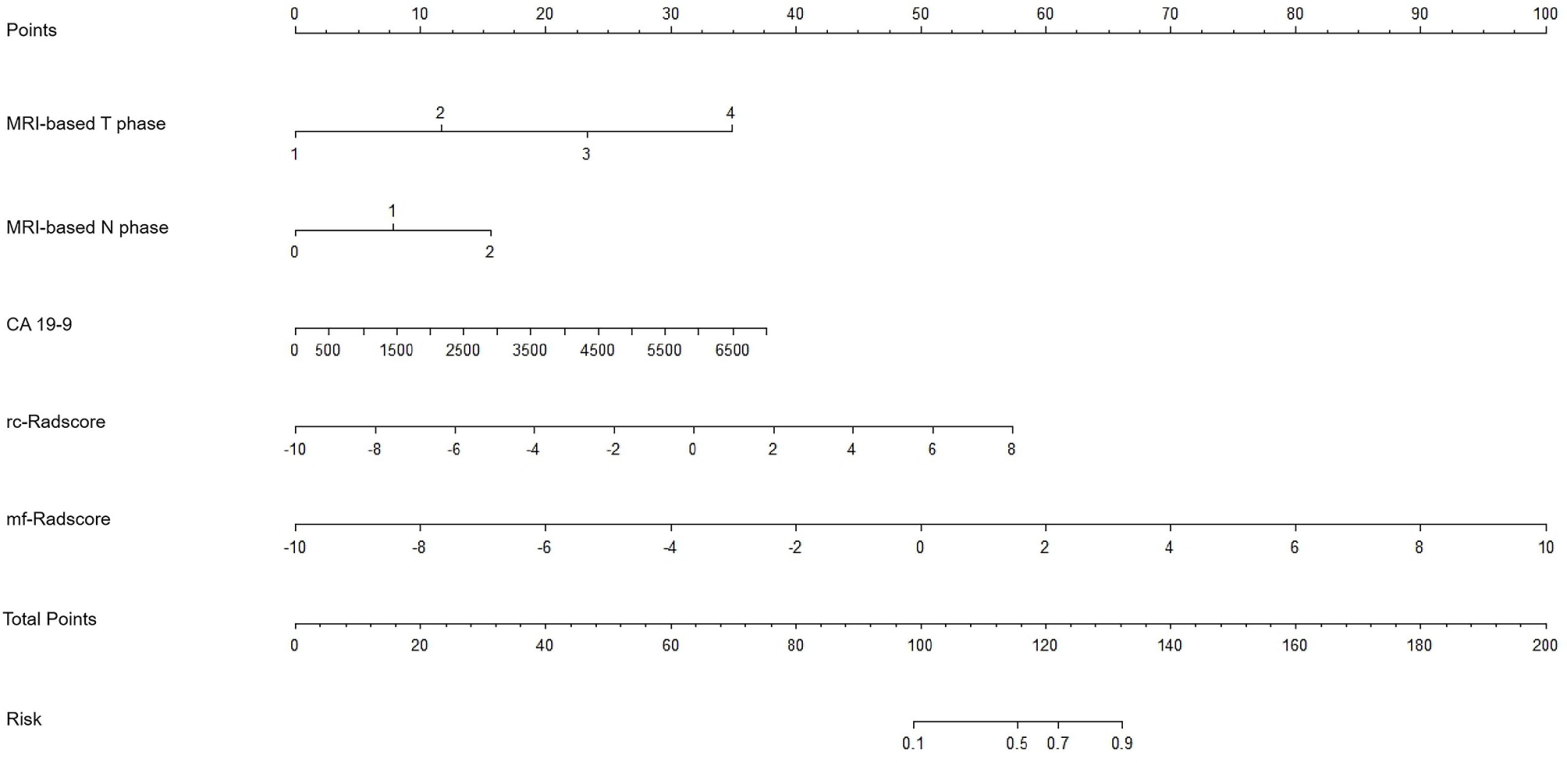
The nomogram of nomo-cMRI/rcRadio/mfRadio.

A nomogram integrated the variables of MRI-based T phase, MRI-based N phase, CA 19 − 9, rc-Radscore, and mf-Radscore (nomo-cMRI/rcRadio/mfRadio) was developed to predict the CRM status of RC (Fig. 3). The AUC of this nomo-cMRI/rcRadio/mfRadio was 0.942 (95%CI, 0.901 to 0.969) in the training set, and 0.909 (95%CI, 0.830 to 0.959) in the testing set, which was slightly superior to that of Logistic-rcRadio and Logistic-mfRadio (Table 3). When considering the integral cohort, the model maintained a high diagnostic performance with an AUC of 0.933 (95%CI, 0.899 to 0.959), achieving a sensitivity of 80.3% and a specificity of 91.5%. A comparison of AUCs across the entire dataset was displayed in Fig. 4.

**Table 3:** The comparison of AUCs of predictive models

	Training set			Testing set		
	AUC (95%CI)	Sensitivity	Specificity	AUC (95%CI)	Sensitivity	Specificity
Logistic-rcRadio	0.883 (0.832 to 0.923)	69.6%	91.5%	0.725 (0.621 to 0.813)	75.0%	66.2%
Logistic-mfRadio	0.891 (0.841 to 0.930)	80.4%	86.6%	0.820 (0.726 to 0.893)	55.0%	98.59%
Nomo-cMRI/rcRadio/mfRadio	0.942 (0.901 to 0.969)	82.6%	93.3%	0.909 (0.830 to 0.959)	95.0%	76.1%

## Discussion

CRMs is defined as the non-peritonealized surface of the excised specimen and is formed during surgery by mesorectal dissection of inferior surface. As early as the 1980s, the distance from the outermost layer of tumor or lymph node deposits in a section to the resection margin was measured microscopically. RCs with involved or threatened CRM carry a particularly high risk of local recurrence. Intraoperative chemoradiotherapy can escalate treatment at the threatened or involved margin at the time of surgery [12]. A previous preoperative staged the RC with MRI found it could accurately predict the potential of i-CRM status, which may help determine patients’ required neo-adjuvant therapy [13]. Preoperative knowledge of the relevant CRM of RCs is essential for neoadjuvant treatment planning and prediction of local recurrence. Compared with patients with lymph node metastasis, patients with CRM involvement by direct tumor spread had a poorer over survival. And the status of CRM could predict prognosis in RC patients with surgery alone, preoperative radiotherapy, and preoperative chemoradiotherapy. After investigating the accuracy of preoperative T-stage, N-stage, and involvement of mesorectal fascia as determined by MRI compared with the histopathological results, it showed that the accuracy of preoperative MRI was lower than expected, which may lead to potentially harmful neoadjuvant treatment or otherwise higher risk of tumor recurrence [14]. According to the study by Jonathan et al., MRI had an accuracy of 63.8% in identifying the i-CRM status of RC, with margin proximity overestimated by 0.4 cm on average [15]. The incidence of i-CRM in our hospital over the past five years was below 8.3%, as there have been over 800 cases of rectal carcinoma pathologically confirmed post-surgically, aligning closely with the previously reported figure of 7.8% [16]. In order to reduce the inaccuracy caused by differences in sample sizes between the two groups, we selected 66 i-CRM patients with mean age of 63.44 ± 13.06 years old and 235 ni-CRM patients with mean age of 64.57 ± 10.80 years old with matched ages for this study. In our analysis of conventional MRI characteristics, we analyzed the characteristics of diameter, distance to anus, MRI-based T/N phase, and clinical information of gender, age, CEA, and CA19-9 to develop a Logistic-cMRI to predict the i-CRM status of RC. Consistent with previous studies, our conventional MRI analysis showed an AUC of 0.864 (95%CI, 0.820 to 0.901) for Logistic-cMRI. Overestimation or understage of CRM in conventional MRI assessment may render some radical surgeries unnecessary [17], and radiologists should be more cautious in interpreting RCs with these characteristics and acknowledge uncertainties in reports.

Therefore, increasing the accuracy of preoperative evaluation of RC is important for optimizing treatment recommendations. Radiomics extracts a large number of quantitative features and has potential for decision-making. Prior studies have suggested that MRI-based radiomics had potential to noninvasively evaluate the biological characteristics of RC [18], as well as to reliably predict response to neoadjuvant therapy in local advanced RC [19]. To the best of our knowledge, there were no relevant studies on MRI-based radiomics for predicting the CRM status of RC. We analyzed not only the MRI-based radiomics of rectal cancer, but also that of mesorectal fascia to predict the CRM status of RC. Both the radiomics of rectal cancer and mesorectal fascia had similar AUC values in prediction. Although there was no article focused on the radiomics assessment of CRM status, it has been demonstrated that MRI-based radiomics features of mesorectal fat can predict pathological complete response and recurrence, as well as post-treatment T and N stage [15]. Our results implied that efficacy of the MRI radiomics features of both rectal cancer and mesorectal fascia were comparable to conventional MRI analysis in predicting the CRM status of RC. Once i-CRM occurs, RC is no longer a local disease, and thus, systemic treatment including chemotherapy and radiotherapy is crucial for these patients. Preoperative assessment of i-CRM status strongly recommends systematic therapy including chemotherapy and radiotherapy for such patients to provide a solid basis for improving disease-free survival and pathological complete response rate [20].

At present, scarcely few articles have conducted the application of radiomics to diagnose the i-CRM status of RC. Prior studies from the literature have shown that MRI-derived radiomics features were reliable in identifying the T stage of RC [21]. To better evaluate the CRM status of RC, radiomics features of tumor and mesorectal fascia were integrated with conventional MRI characteristics into a nomogram. Theoretically, the nomo-cMRI/rcRadio/mfRadio owed the highest AUC values in both the training set (0.942 vs. 0.883 and 0.891) and the testing set (0.909 vs. 0.725 and 0.820). This promising result attributed to the complementary function of conventional MRI characteristics, radiomics feature of rectal cancer and mesorectal fascia. Some investigators have reported that preoperative T phase was an independent risk factor related to CRM status, which affected local recurrence rates and overall survival rates [22]. Hence, our comprehensive analysis containing the variables of MRI-based T phase, MRI-based N phase, rc-Radscore, and mf-Radscore is of paramount significance in predicting the CRM status of RC. MRI, with its excellent soft tissue resolution on multiple planes and the ability to distinguish the three-layer structure of the bowel wall and adjacent rectal mesentery fascia without ionizing radiation, can objectively quantify images into a large amount of data through radiomics analysis, making it an important method for better evaluating RC.

This study has the following shortcomings. First, with consideration of the excellent interobserver agreement for MRI-based radiomics features in RC [23], the VOI-rc and VOI-mf were depicted by two radiologists in consultation. Second, we manually delineated the VOI, though the effort of coregistration of different sequences was made to match images. However, automatic segmentation of MRI images of RC using a U-net algorithm achieved a higher accuracy, sensitivity, and specificity than manual segmentation to predict the response to treatment in local advanced rectal cancer [24]. Thus, the automatic segmentation of RC to evaluate the CRM status should also be considered for further study. Third, we analyzed all of the T1WI, T2WI, DWI, and T1CE sequences as a whole to construct predictive models in evaluating the CRM status of RC. Although our results were encouraging, the different behavior of separate sequences deserves further investigation. As a future step, we will focus on the utilization of automatic segmentation and subdivision of different MRI sequences to explore their correlation with pathology.

## Conclusion

In conclusion, our study demonstrated that MRI-based radiomics of both rectal cancer and mesorectal fascia have the potential to predict the CRM status of RC, which helps guide personal treatment.

### Electronic supplementary material

Below is the link to the electronic supplementary material.

Supplementary Material 1

## Data Availability

All data generated or analyzed during this study are included in this article.

## Abbreviations

RC: Rectal cancer
TME: Total mesorectal excision
MRI: Magnetic resonance imaging
i-CRM: CRM involvement
ni-CRM: Without CRM involvement
CEA: Carcinoma Embryonic Antigen
CA 19 − 9: Carbohydrate antigen 19 − 9
VOI: Volume of interest
VOI-rc: VOI of rectal cancer
VOI-mf: VOI of mesorectal fascia
SMOTE: Synthetic minority over-sampling technique
RC: GBDT: Gradient boosting decision tree
Logistic-cMRI: Logistic model of conventional MRI
Logistic-rcRadio: Logistic model of radiomics of rectal cancer
Logistic-mfRadio: Logistic model of radiomics of mesorectal fascia
ROC Area under curve AUC: Receiver operator characteristics curve
Nomo-cMRI/rcRadio/mfRadio: Nomogram integrated conventional MRI and radiomics
