# Supplementary material for: Comparison of conventional MRI analysis versus MRI-based radiomics to predict the circumferential margin resection involvement of rectal cancer
Source: BMC Gastroenterol. 2024 Jun 20;24:209. doi: 10.1186/s12876-024-03274-z (PMC11191295; doi:10.1186/s12876-024-03274-z)
Supplement: Supplementary file 1 — Supplementary Material 1 [file 12876_2024_3274_MOESM1_ESM.doc]

**Supplementary material**

**Section 1-conventional MRI analysis:** the conventional MRI characteristics included the diameter of rectal cancer, the distance of tumor to anus, MRI-based T phase, and MRI-based N phase. The diameter of rectal cancer was measured the long diameter of the lesion. The distance to anus was measured from the lower margin of tumor to the anus. MRI-based T phase classifies the growth of the tumor into the rectal wall and its spread into the mesorectum and adjacent structures. At MRI, the muscularis propria appears as a thin black line encircling the outer wall of the rectum, and tumor extension through this line indicates T3 disease. Further tumor extension into adjacent organs indicates T4 disease^[1]^. T1 tumors are located in the submucosa and T2 tumors are infiltrating the muscularis propia. And there is difficulty in distinguishing between T1 and T2 phase tumors. MRI-based N phase refers to the evaluation of tumor involvement of regional lymph nodes, i.e. perirectal lymph nodes as well as lymph nodes along the arteries supplying the rectum. In general, all locoregional nodes with a short axis greater than 9 mm in length should be considered suspicious. Nodes with a short axis that are less than 9 mm in length should meet certain morphological or imaging criteria before being considered suspicious. The 3 major criteria include an irregular or indistinct outline, heterogeneous T2W signal intensity, and a round shape. All 3 criteria should be met for a locoregional node measuring less than 5 mm in length. Nodes measuring between 5 and 9 mm should meet at least 2 criteria^[2]^.

**Section 2-VOI segmentation:** Before segmentation, the images were preprocessed by a software of Matlab (Version 9.6) in order to matches all the scanned images into standard spatial coordinate system. The images of T1WI, DWI, and T1CE were registered to those of T2WI by selecting the Estimate & reslice option of the Coregister module in SPM 12 of Matlab software (sFigure 1). Then, the VOI-rc and VOI-mf were manually delineated using a software of ITK-SNAP in T2WI images with the consensus between two radiologists (10- and 12-years’ experience of MR diagnosis, Doctor Ma and Doctor Guan). The VOIs of T2WI were loaded into T1WI, DWI, and T1CE sequences, after manually fine-tuned, the relevant VOIs were acquired.


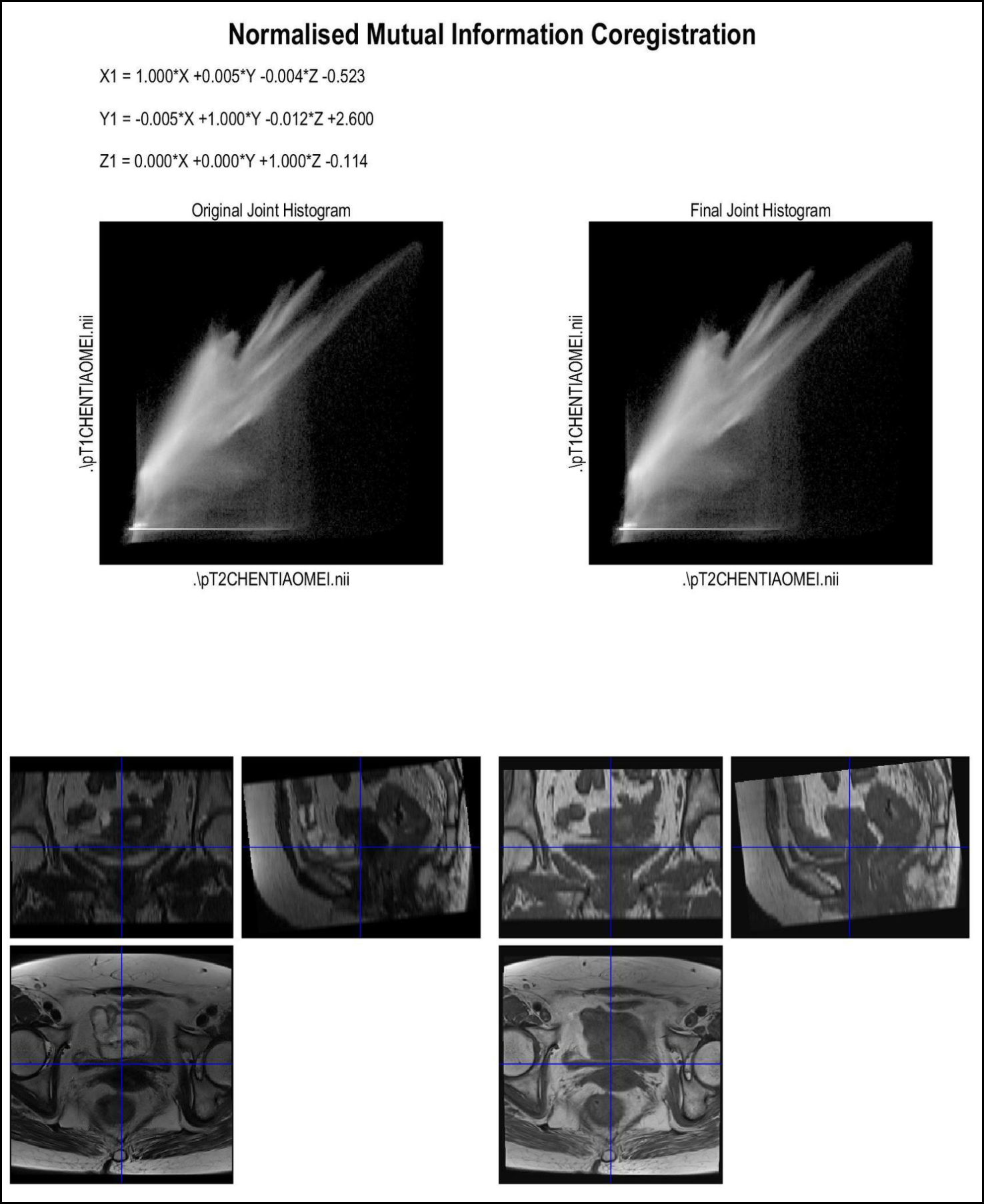


**sFigure 1.** The information of coregistration of different MRI sequences.

**Section 3-Radiomics features:** The radiomics features in this article calculated by A.K. software included first order statistics features, shape-based (3D) features, gray level run length matrix features (GLRLM), gray level size zone matrix features (GLSZM), gray level co-occurrence matrix features (GLCM), neighbouring gray tone difference matrix features (NGTDM), and gray level dependence matrix features (GLDM).

First-order statistics features describe the distribution of voxel intensities within the image region defined by the mask through commonly used and basic metrics. They were Energy, Total Energy, Entropy, Minimum, 10th percentile, 90th percentile, Maximum, Mean, Median, Interquartile Range, Range, Mean Absolute Deviation (MAD), Robust Mean Absolute Deviation (rMAD), Root Mean Squared (RMS), Standard Deviation, Skewness, Kurtosis, Variance, Uniformity. Shape-based (3D) features we included descriptors of the three-dimensional size and shape of the ROI. These features are independent from the gray level intensity distribution in the ROI and are therefore only calculated on the non-derived image and mask. GLRLM features quantifies gray level runs, which are defined as the length in number of pixels, of consecutive pixels that have the same gray level value. GLSZM features quantifies gray level zones in an image. A gray level zone is defined as a the number of connected voxels that share the same gray level intensity. GLCM features describes the second-order joint probability function of an image region constrained by the mask and is defined as P(i, j|δ,θ). NGTDM features quantifies the difference between a gray value and the average gray value of its neighbors within distance δ. The sum of absolute differences for gray level i is stored in the matrix. GLDM features quantifies gray level dependencies in an image. A gray level dependency is defined as a the number of connected voxels within distance δ that are dependent on the center voxel.

**Section 4-Radiomics feature selection:** After replacing the outlier variables by median, the method of standardization was used. Standardization: Extracted radiomics features were standardized, which removed the unit limits of the data of each feature and converted it into a dimensionless pure value. This allowed the indexes of different units or orders to be compared and weighted. We used a z-score normalization to make the image intensities fit a standard normal distribution with μ=0 andσ=1, whereμis the mean value of the images, andσis the standard deviation. The normalized values (also called z-scores) of the image intensities (χ) were calculated as follow:


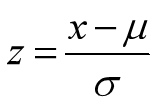


Then the analysis of variance and correlation analysis were utilized to select features. The correlation test was calculated to reduce data redundancy. The software calculated the paired correlation between each two of the features. If the correlation coefficient was greater than 0.5, which showed that the two features were highly correlated, one of them was removed. Third, the method of gradient boosting decision tree (GBDT) was used to select radiomic features. Gradient boosting decision tree (GBDT) is a Boosting algorithm based on decision tree as base learner. It builds a decision tree in each iteration to reduce the residual of the current model in the gradient direction. GBDT is commonly used for regression, classification and feature selection. GBDT’s advantages include: (a) It flexible processes of various types of data, including both continuous and discrete data set; (b) It has powerful predictive ability and generalization ability; (c) It has good interpretability, and robustness, can automatically discover high-order relationships between features, and does not require data normalization and other processing^[3]^.

**Reference**

1. Moreno CC, Sullivan PS, Mittal PK. MRI Evaluation of Rectal Cancer: Staging and Restaging. Curr Probl Diagn Radiol. 2017;46(3):234-241.

2. Bates DDB, Homsi ME, Chang KJ, Lalwani N, Horvat N, Sheedy SP. MRI for Rectal Cancer: Staging, mrCRM, EMVI, Lymph Node Staging and Post-Treatment Response. Clinical colorectal cancer. 2022;21(1):10-18.

3. Liang Y, Zhang S, Qiao H, Cheng Y. iEnhancer-MFGBDT: Identifying enhancers and their strength by fusing multiple features and gradient boosting decision tree. Mathematical biosciences and engineering : MBE. 2021;18(6):8797-8814.
